# Access to Firearms and Opioids Among Veterans at Risk for Suicide

**DOI:** 10.1001/jamanetworkopen.2024.56906

**Published:** 2025-01-28

**Authors:** Gabriela Kattan Khazanov, Matthew Wilson, Tom Cidav, Christopher B. Roberts, Catherine Barry, James R. McKay, Shari Jager-Hyman, Marianne Goodman, Joseph Simonetti

**Affiliations:** 1 Center of Excellence for Substance Addiction Treatment and Education, Corporal Michael J Crescenz VA Medical Center, Philadelphia, Pennsylvania; 2 University of Pennsylvania Perelman School of Medicine, Philadelphia; 3 Center for Health Equity Research and Promotion, Corporal Michael J Crescenz VA Medical Center, Philadelphia, Pennsylvania; 4 Program Evaluation and Resource Center, Offices of Mental Health and Suicide Prevention, Palo Alto VA Medical Center, Palo Alto, California; 5 Mental Illness Education and Research Center, JJ Peters VA Medical Center, Bronx, New York; 6 Mount Sinai School of Medicine, New York, New York; 7 VA Rocky Mountain Mental Illness Research, Education and Clinical Center for Suicide Prevention, Rocky Mountain Regional VA Medical Center, Aurora, Colorado; 8 Firearm Injury Prevention Initiative, University of Colorado Anschutz School of Medicine, Aurora

## Abstract

**Importance:**

Firearm injury and poisoning, often by drug or medication overdose, account for most suicides among the general population and US veterans. In the Veterans Health Administration, the largest integrated health care system in the US, firearm and opioid access is assessed among patients at risk for suicide who complete suicide safety plans.

**Objective:**

To describe self-reported, clinician-documented access to firearms and opioids, firearm storage practices, distribution of firearm cable locks and naloxone, and counseling on firearm storage and overdose among veterans at elevated risk for suicide who completed suicide safety plans.

**Design, Setting, and Participants:**

This cross-sectional study used electronic health record data from the Veterans Health Administration from December 2021 to February 2023. Participants were veterans identified as having elevated suicide risk through routine screening with the Columbia Suicide Severity Rating Scale Screener who completed a safety plan within 30 days. Data were analyzed from March 2023 to March 2024.

**Exposure:**

Completion of a safety plan, a brief, evidence-based intervention to help prevent or de-escalate suicidal crises.

**Main Outcomes and Measures:**

Firearm and opioid access, as well as firearm storage information, were assessed via the lethal means component of the standardized safety plan note template.

**Results:**

Among 38 454 veterans identified (32 310 [84.0%] male; 15 206 participants [39.5%] aged ≥55 years; 26 960 participants [70.1%] living in urban areas), 9969 (25.9%) were Black and 23 714 (61.7%) were White and 3426 (8.9%) were Hispanic/Latine and 28 892 (75.1%) were not Hispanic/Latine. A total of 10 855 (28.2%) reported access to firearms. Approximately one-third of veterans reported storing at least 1 firearm in each of the following ways: unlocked and loaded (insecure), outside of the home or locked and unloaded (secure), or locked and loaded. Younger and middle-aged veterans, White veterans, veterans who were not Hispanic/Latine, male veterans, and rural veterans were more likely to report firearm access. A total of 2021 veterans (5.3%) reported access to opioids; older veterans, White veterans, veterans who were not Hispanic/Latine, and rural veterans were more likely to report opioid access. Clinicians reported discussing firearm storage with 10 655 veterans (98.2%) and overdose with 1589 veterans (78.6%). Only 1837 veterans (16.9%) offered firearm cable locks and 536 veterans (26.5%) of veterans offered naloxone were documented as accepting them.

**Conclusions and Relevance:**

In this cross-sectional study of electronic health record data, the prevalence of reported access to firearms was lower than expected, suggesting underreporting or underdocumentation, or a lower true prevalence among this at-risk population. Completion of a note template may have encouraged routine discussion of firearm storage and overdose risk, but acceptance of gunlocks and naloxone was low. These findings suggest that White veterans, veterans who were not Hispanic/Latine, and rural veterans may be at particular risk of harm by firearms and opioids.

## Key Points

**Question:**

Among veterans at risk for suicide receiving suicide safety plans in the Veterans Health Administration, what is the prevalence of reported access to firearms and opioids and implementation of related interventions?

**Findings:**

In this cross-sectional study of 38 454 veterans at risk for suicide, 28% reported access to firearms and 5% reported access to opioids. As documented by clinicians in a note template, firearm storage and overdose were discussed with 79% to 98% of veterans, but relatively few accepted firearm cable locks and/or naloxone.

**Meaning:**

These findings suggest that completion of a note template may encourage routine discussion of risks related to firearms and opioids, although reported access to firearms was lower than expected.

## Teaser

This cross-sectional study among veterans at elevated risk for suicide who completed suicide safety plans describes self-reported, clinician-documented access to firearms and opioids, firearm storage practices, distribution of firearm cable locks and naloxone, and counseling on firearm storage and overdose.

## Introduction

Suicide is a leading cause of death in the US, particularly among veterans.^1^ Firearm injury and poisoning, often by drug or medication overdose, account for most suicide deaths among the general population (52% for firearms and 12% for poisoning) and veterans (72% for firearms and 8% for poisoning). Access to opioids in particular has been associated with increased risk for suicide, with suicides due to opioid overdose nearly tripling over the last 20 years.^2,3^ Firearm access has increased substantially since 2020 and is also strongly associated with suicide risk.^4,5^

Veterans report more access to firearms and less secure storage relative to the general population. In 2022, studies of nationally representative samples found that 51% of veterans owned firearms (up from 45% in 2015), with male and rural veterans reporting higher rates of firearm ownership.^6,7,8^ By contrast, in 2022, approximately one-third of the general US population owned firearms.^5^ Among veteran firearm owners, 30% to 40% reported storing at least 1 firearm loaded and unlocked; male and rural veterans were more likely to report insecure storage practices.^9,10^ Veterans also have high rates of opioid access and opioid overdoses, perhaps due to greater levels of chronic pain and substance use relative to the general population.^11,12^ Approximately 7% of veterans were being prescribed an opioid medication in 2019, which does not account for nonprescribed use of opioids or prescriptions provided outside of the Veterans Health Administration (VHA).^13^ In addition, 0.7% of veterans met criteria for an opioid use disorder in 2021, and opioid overdoses increased from 15 to 21 per 100 000 population from 2010 to 2016.^14,15^ Studies have found that White veterans, veterans who are not Hispanic/Latine, and rural veterans report greater access to opioids than veterans identifying with other races or ethnicities and those in urban settings.^16,17^

In response to these trends, the VHA, the largest integrated health care system in the US, has made significant efforts to educate veterans about the risks of access to firearms and opioids, as well as the benefits of secure firearm storage and harm reduction strategies for those with opioid access. For example, the VHA has disseminated information about how to respond to an opioid overdose and distributed naloxone.^12,18^ Efforts also include training clinicians to assess access to lethal means and recommend safety practices (ie, lethal means counseling [LMC]), and embedding LMC in other suicide prevention interventions, like suicide safety planning. Suicide safety planning is a brief, evidence-based intervention that helps patients identify strategies to prevent or de-escalate suicidal crises.^19,20^ Safety planning includes 6 steps, the last of which is LMC, a standalone, evidence-based intervention.^21^ During the LMC component of safety planning, clinicians assess veterans’ access to firearms and opioids and storage of firearms; offer firearm locking devices, as well as overdose education and naloxone; and work with veterans to develop strategies to limit their access to firearms and opioids during periods of higher suicide risk. Clinicians are encouraged to discuss limiting access to any other potentially lethal mean identified by the veteran (eg, other toxic substances, ligatures), but do not complete standardized prompts for these means.

Prior work has begun to evaluate access to and storage of firearms among veterans at elevated risk for suicide. One study found that 45% of veterans receiving mental health services across 5 VHA facilities reported access to firearms; 47% of those with suicidal thoughts and 56% of those with a suicide plan endorsed firearm access.^10^ Another study in a nationally representative sample found that firearm storage practices were similar among veterans with and without self-reported risk factors for suicide.^9^ These studies did not assess access to firearms and opioids or implementation of interventions to improve secure firearm storage and limit overdoses among a national sample of veterans at elevated risk for suicide in the VHA. This information is critical to informing suicide prevention efforts in the VHA and other large health systems considering the implementation of similar interventions.

In this study, we use national electronic health record (EHR) data for veterans with elevated suicide risk who completed safety plans to examine: (1) reported access to firearms, firearm storage practices, clinician-led counseling on firearm storage practices, and distribution of firearm locking devices; (2) reported access to opioids, distribution of naloxone, and delivery of overdose education; and (3) sociodemographic characteristics associated with reported access to firearms and opioids, specific firearm storage practices, and acceptance of firearm locks and naloxone. Based on previous research, we hypothesized that approximately one-half of veterans would report access to firearms, one-third of veterans with access to firearms would report storing their firearms insecurely (eg, loaded and unlocked), and that male and rural veterans would report more firearm access. We also hypothesized that White and rural veterans would report greater access to opioids. Other analyses were exploratory.

## Methods

### Population

The institutional review board of the Philadelphia VA Medical Center approved this study and provided a waiver of informed consent because because analyses were based on electronic health records. We followed the Strengthening the Reporting of Observational Studies in Epidemiology (STROBE) reporting guideline for cross-sectional studies.

All veterans in the VHA are screened at least annually, and as indicated, with the Columbia Suicide Severity Rating Scale Screener (C-SSRS),^22^ a standardized, 8-item, clinician-administered suicide risk screening tool. We followed VHA guidelines in defining veterans at elevated risk for suicide as those with plans or intent to attempt suicide over the previous month or suicidal behavior in the previous 3 months (eAppendix 1 in Supplement 1). We examined responses to the LMC component of the suicide safety plan (step 6) among veterans at elevated risk for suicide who completed a safety plan within 30 days of being screened. We extracted standardized EHR note template data from December 2021, when substantial changes to the LMC section of the template were adopted by all VHA sites, through February 2023.

Safety planning is strongly recommended for veterans at elevated risk for suicide across settings and is specifically mandated for 2 groups. First, safety planning is required in acute care settings (the emergency department or urgent care) for veterans who have positive screening results for elevated suicide risk on the C-SSRS, are subsequently determined to be at intermediate or high acute or chronic risk of suicide, and are discharged home (referred to as the *acute care group*). Second, safety planning is required for veterans flagged by clinicians as being at particularly high risk for suicide, and the veteran is then followed-up by the hospital’s suicide prevention team (referred to as the *flagged group*).

### Measures

Data extracted from the LMC component of safety plans included clinician-documented responses to prompts that evaluated veterans’ access to firearms and opioids and storage of firearms, clinicians’ counseling on firearm storage and overdose education, and distribution of firearm locks and naloxone. Prompts and response options are provided in Table 1. Except for the firearm storage prompt, clinicians could only select 1 response. For the firearm storage prompt, clinicians were asked to select all applicable responses, resulting in some seemingly inconsistent responses (eg, loaded with ammunition and unloaded and stored with ammunition). We categorized these inconsistent responses hierarchically, prioritizing the most secure response option endorsed, and grouped storage options into the following categories, based on previous research^9,23^: (1) outside of the home, (2) locked and unloaded, (3) locked and loaded, and (4) unlocked and loaded (Table 1). Sociodemographic categories, including race and ethnicity, were self-reported and captured in the EHR. Participants identified as Black, White, or other (including American Indian/Pacific Islander, Asian, and unknown) race and Hispanic/Latine, not Hispanic/Latine, or unknown ethnicity. Race and ethnicity were assessed to examine sociodemographic differences in responses. Rural vs urban location was based on veterans’ rural-urban commuting area codes.^24^

**Table 1.** Prompts, Response Options, and Categories Derived From the Lethal Means Counseling Component of the Suicide Safety Plan^a^

Construct	Prompt	Response options	Response groupings
Access to firearms	Do you have access to firearms in your home or elsewhere? (eg, off site storage, through work, friends, or family)	No, yes	NA
Firearm storage [For those with access to firearms]^b^	How do you store your firearms? Do you have any that are stored (select all that apply):	Locked with an external device, such as a cable lock or trigger lock Locked in a gun safe or lockbox Unlocked (eg, not using either an external device or using a gun safe) Loaded with ammunition Unloaded and stored separately from ammunition Unloaded and stored with ammunition Disassembled or nonfunctional Outside of my immediate home and property (eg, with a friend/family, personal storage unit, shooting range, gun shop, with law enforcement, pawn shop) Other	In hierarchical order: Outside of home/property (E) Locked (A or B) and unloaded (E or F) Locked (A or B) and loaded (D or no response to E or F; D prioritized if clinician selected D and E or F) Unlocked (C or no response to A or B; C prioritized if clinician selected C and A or B) and loaded (D or no response to E or F; D prioritized if clinician selected D and E or F) All others
Discussion of firearm storage (dropdown for those indicating access to firearms)	Firearm safe storage discussed with the veteran	Yes, no	NA
Distribution of firearm locking devices (dropdown for those indicating access to firearms)	Ask the veteran “Would you like any gunlocks?” Choose only one.	No, patient declined No, provider [clinician] did not offer Yes, and gunlock(s) provided Veteran requests gunlock(s) but they are not presently available N/A; other safe storage options in place	Patient declined or NA or other safe storage option in place (A or E) No, clinician did not offer (B) Yes, and gunlock(s) provided (C) Veteran requested gunlock(s) but they are not presently available (D)
Access to opioids	Do you have access to opioids?	No, Yes	NA
Overdose education (dropdown for those indicating access to opioids)	Did you discuss safety and provide overdose education with the Veteran including the use of naloxone?	Education could not be provided; education provided	NA
Distribution of naloxone (dropdown for those indicating access to opioids)	Naloxone. Choose one:	Order naloxone Provider [clinician] notified of request for naloxone Has current naloxone (ie, not used and not expired) Patient declined naloxone Other	Order naloxone or clinician notified of request for naloxone (A or B; combined because both indicate that the clinician requested naloxone for the veteran) Has current naloxone (ie, not used and not expired; C) Patient declined naloxone (D) Other (E)

Abbreviation: NA or N/A, not applicable.

^a^ Prompts and response options are taken verbatim from the Safety Planning note template.

^b^ Inconsistent responses were categorized hierarchically, in the order indicated in response groupings.

### Statistical Analysis

Among veterans who completed safety plans within 30 days of being identified as at elevated risk for suicide on the C-SSRS, we describe responses to each LMC prompt among all veterans and among sociodemographic subgroups (Table 1). We then describe sociodemographic differences based on veterans’ firearm access, opioid access, firearm storage, and distribution of firearm locks and naloxone by conducting χ^2^ tests, followed by logistic regressions using odds ratios (ORs) and 95% CIs to examine differences among subgroups. We repeated each analysis controlling for other sociodemographic factors and then controlling for both other sociodemographic factors and veterans’ inclusion in the acute care or flagged groups. *P* values were 2-sided, and statistical significance was set at the 95th percentile. Analyses were conducted using SAS software version 9.4 (SAS Institute). Data were analyzed from March 2023 to March 2024.

## Results

We identified 38 454 veterans (32 310 [84.0%] male; 15 206 participants [39.5%] aged ≥55 years; 26 960 participants [70.1%] living in urban areas) at elevated risk for suicide who completed safety plans within 30 days of being identified as at risk; 9969 (25.9%) were Black and 23 714 (61.7%) were White and 3426 (8.9%) were Hispanic/Latine and 28 892 (75.1%) were not Hispanic/Latine (Table 2). Of these veterans, 3127 (8.1%) were in the acute care group, 13 971 (36.3%) were in the flagged group, and 15 915 (41.4%) were in either of the 2 groups (Table 3).

**Table 2.** Reported Prevalence of Access to Firearms, Opioids, and Related Interventions Among Veterans at Elevated Risk for Suicide Who Completed Safety Plans

Measure	Veterans, No. (%)														
	Overall (N = 38 454)	Sex		Race					Ethnicity		Age, y			Residence	
		Male (n = 32 310)	Female (n = 6144)	American Indian/Pacific Islander (n = 1086)^a^	Asian (n = 752)	Black (n = 9969)	White (n = 23 714)	Unknown (n = 2933)	Hispanic/Latine (n = 3426)	Not Hispanic/Latine (n = 28 892)	18-39 (n = 12 859)	40-54 (n = 10 389)	≥55 (n = 15 206)	Urban (n = 26 960)	Rural (n = 9729)^b^
**Firearm access**															
Access to firearms^c^	10 855 (28.2)	9366 (29.0)	1489 (24.2)	279 (25.7)	203 (27.0)	2337 (23.4)	7140 (30.1)	896 (30.5)	844 (24.6)	8074 (27.9)	3833 (29.8)	3097 (29.8)	3925 (25.8)	6886 (25.5)	3651 (37.5)
Firearm storage^d^															
Outside of the home	1185 (10.9)	1069 (11.4)	116 (7.8)	31 (11.1)	23 (11.3)	243 (10.4)	795 (11.1)	93 (10.4)	90 (10.7)	884 (10.9)	467 (12.2)	321 (10.4)	397 (10.1)	761 (11.0)	376 (10.3)
Locked and unloaded	1866 (17.2)	1636 (17.5)	230 (15.4)	41 (14.7)	51 (25.1)	351 (15.0)	1253 (17.5)	170 (19.0)	167 (19.8)	1332 (16.5)	707 (18.4)	574 (18.5)	585 (14.9)	1168 (17.0)	642 (17.6)
Locked and loaded	4406 (40.6)	3746 (40.0)	660 (44.3)	120 (43.0)	77 (37.9)	967 (41.4)	2875 (40.3)	367 (41.0)	339 (40.2)	3220 (39.9)	1607 (41.9)	1366 (44.1)	1433 (36.5)	2871 (41.7)	1409 (38.6)
Unlocked and loaded	2989 (27.5)	2560 (27.3)	429 (28.8)	72 (25.8)	32 (15.8)	674 (28.8)	1961 (27.5)	240 (26.8)	214 (25.4)	2337 (28.9)	897 (23.4)	748 (24.2)	1344 (34.2)	1854 (26.9)	1057 (29.0)
Other	219 (2.0)	193 (2.1)	26 (1.7)	NA	NA	45 (1.9)	147 (2.1)	15 (1.7)	19 (2.3)	158 (2.0)	79 (2.1)	40 (1.3)	100 (2.5)	129 (1.9)	85 (2.3)
Missing	190 (1.8)	162 (1.7)	28 (1.9)	NA	NA	57 (2.4)	109 (1.5)	11 (1.2)	15 (1.8)	139 (1.7)	76 (2.0)	48 (1.5)	66 (1.7)	103 (1.5)	82 (2.2)
Firearm-related interventions^d^															
Safe firearm storage discussed	10 665 (98.2)	9040 (96.5)	1445 (97.0)	270 (96.8)	194 (95.6)	2238 (95.8)	6913 (96.8)	870 (97.1)	816 (96.7)	7792 (96.5)	3704 (96.7)	2995 (96.7)	3786 (96.5)	6660 (96.7)	3519 (96.4)
Gunlocks not offered	382 (3.5)	327 (3.5)	55 (3.6)	NA	11 (5.4)	85 (3.6)	251 (3.5)	29 (3.2)	27 (3.2)	285 (3.5)	143 (3.7)	107 (3.5)	132 (3.4)	246 (3.6)	129 (3.5)
Gunlocks provided	1837 (16.9)	1557 (16.6)	280 (18.8)	52 (18.6)	33 (16.3)	536 (22.9)	1058 (14.8)	158 (18.7)	162 (19.2)	1331 (16.5)	642 (16.7)	488 (15.8)	707 (18.0)	1196 (17.4)	592 (16.2)
Gunlocks declined	8239 (75.9)	7159 (76.4)	1080 (72.5)	206 (73.8)	146 (71.9)	1604 (68.6)	5602 (78.5)	681 (76.0)	623 (73.8)	6162 (76.3)	2903 (75.7)	2405 (77.7)	2931 (74.7)	5204 (75.6)	2786 (76.3)
Gunlocks requested but none available	241 (2.2)	190 (2.0)	51 (3.4)	NA	NA	63 (2.7)	141 (2.0)	19 (2.1)	19 (2.3)	173 (2.1)	91 (2.4)	57 (1.8)	93 (2.4)	157 (2.3)	75 (2.1)
Missing gunlocks data	158 (1.5)	135 (1.4)	23 (1.5)	NA	NA	49 (2.1)	90 (1.3)	NA	13 (1.5)	121 (1.5)	54 (1.4)	41 (1.3)	63 (1.6)	84 (1.2)	70 (1.9)
**Opioid access**															
Access to opioids^c^	2021 (5.3)	1681 (5.2)	340 (5.5)	43 (4.0)	32 (4.3)	434 (4.4)	1368 (5.8)	144 (4.9)	131 (3.8)	1690 (5.8)	388 (3.0)	496 (4.8)	1137 (7.5)	1324 (4.9)	617 (6.3)
Opioid-related interventions^e^															
Safety and overdose education provided	1589 (78.6)	1318 (78.4)	271 (79.7)	33 (76.7)	25 (78.1)	339 (78.1)	1081 (79.0)	111 (77.1)	99 (75.6)	1326 (78.5)	304 (78.4)	396 (80.0)	889 (78.2)	1033 (78.0)	497 (80.1)
Naloxone ordered	536 (26.5)	444 (26.4)	92 (27.1)	14 (32.6)	NA	116 (26.7)	355 (26.0)	42 (29.2)	40 (30.5)	447 (26.4)	107 (27.6)	136 (27.4)	293 (25.8)	355 (26.8)	157 (25.4)
Patient had naloxone already	370 (18.3)	315 (18.7)	55 (16.2)	NA	NA	71 (16.6)	268 (19.6)	18 (12.5)	20 (15.3)	321 (19.0)	39 (10.1)	100 (20.2)	231 (20.3)	231 (17.4)	128 (20.7)
Patient declined naloxone	542 (26.8)	455 (27.1)	87 (25.6)	12 (27.9)	13 (40.6)	120 (27.6)	362 (26.5)	35 (24.3)	28 (21.4)	442 (26.2)	111 (28.6)	123 (24.8)	308 (27.1)	347 (26.2)	174 (28.2)
Other naloxone response	550 (27.2)	449 (26.7)	101 (29.7)	11 (25.6)	NA	128 (29.5)	358 (26.2)	47 (32.6)	43 (32.8)	462 (27.3)	124 (32.0)	143 (28.8)	283 (24.5)	376 (28.4)	147 (23.8)
Missing naloxone data	23 (1.1)	18 (1.1)	NA	0	0	0	23 (1.0)	NA	0	18 (1.1)	NA	0	22 (1.9)	15 (1.1)	11 (1.8)

Abbreviation: NA, not available (suppressed because count was <10 veterans).

^a^ American Indian/Pacific Islander group includes Alaska Native and Native Hawaiian veterans.

^b^ Rural category includes both Rural and Highly Rural Rural-Urban Commuting Area codes.

^c^ Denominator is the total cohort row.

^d^ Denominator is the number with reported access to firearms.

^e^ Denominator is the number with access to opioids.

**Table 3.** Demographic Differences Among Veterans at Elevated Risk for Suicide With and Without Access to Firearms and Opioids

Demographic group	Access to firearms				Access to opioids			
	Veterans, No. (%)			χ^2^	Veterans, No. (%)			χ^2^
	Total (n = 38 453)^a^	Access (n = 10 855 [28.2%])	No access (n = 27 598 [71.8%])		Total (n = 38 454)	Access (n = 2021 [5.3%])	No access (n = 36 433 [94.7%])	
Age, y								
18-39	12 859 (33.4)	3833 (35.3)	9026 (32.7)	72.53^b^	12 859 (33.4)	388 (19.2)	12 471 (34.2)	284.94^b^
40-54	10 388 (27.0)	3097 (28.5)	7291 (26.4)		10 389 (27.0)	496 (24.5)	9893 (27.2)	
≥55	15 206 (39.5)	3925 (36.2)	11 281 (40.9)		15 206 (39.5)	1137 (56.3)	14 069 (38.6)	
Sex								
Female	6144 (16.0)	1489 (13.7)	4655 (16.9)	57.58^b^	6144 (16.0)	340 (16.8)	5804 (15.9)	1.14
Male	32 309 (84.0)	9366 (86.3)	22943 (83.1)		32 310 (84.0)	1681 (83.2)	30 629 (84.1)	
Race								
Available data, No.	35 520	9959	25 561	NA	35 521	1877	33 644	NA
Black	9969 (28.1)	2337 (23.5)	7632 (29.9)	157.79^b^	9969 (28.1)	434 (23.1)	9535 (28.3)	33.70^b^
White	23 713 (66.8)	7140 (71.7)	16 573 (64.8)		23 714 (66.8)	1368 (72.9)	22 346 (66.4)	
Other^c^	1838 (5.2)	482 (4.8)	1356 (5.3)		1838 (5.2)	75 (4.0)	1763 (5.2)	
Ethnicity								
Hispanic/ Latine	3426 (8.9)	844 (7.8)	2582 (9.4)	58.19^b^	3426 (8.9)	131 (6.5)	3295 (9.0)	83.66^b^
Not Hispanic/ Latine	28 891 (75.1)	8070 (74.3)	20 821 (75.4)		28 892 (75.1)	1690 (83.6)	27 202 (74.7)	
Unknown^c^	6136 (16.0)	1941 (17.9)	4195 (15.2)		6136 (16.0)	200 (9.9)	5936 (16.3)	
Location								
Available data, No.	36 688	10 537	26 151	NA	36 689	1941	34 748	NA
Rural^d^	9729 (26.5)	3651 (34.7)	6078 (23.2)	501.57^b^	9729 (26.5)	617 (31.8)	9112 (26.2)	29.21^b^
Urban	26 959 (73.5)	6886 (65.4)	20 073 (76.8)		26 960 (73.5)	1324 (68.2)	25 636 (73.8)	
Acute care group^e^								
Yes	3127 (8.1)	629 (5.8)	2498 (9.0)	110.61^b^	3127 (8.1)	166 (8.2)	2961 (8.1)	0.02
No	35 326 (91.9)	10 226 (94.2)	25 100 (91.0)		35327 (91.9)	1855 (91.8)	33472 (91.9)	
Flagged group^f^								
Yes	13 971 (36.3)	3432 (31.6)	10 539 (38.2)	145.41^b^	13972 (36.3)	726 (35.9)	13246 (36.4)	0.16^g^
No	24 482 (63.7)	7423 (68.4)	17 059 (61.8)		24482 (63.7)	1295 (64.1)	23187 (63.6)	
Acute care or flagged group^e^^,^^f^								
Yes	15 915 (41.4)	3844 (35.4)	12 071 (43.7)	222.66^b^	15 916 (41.4)	819 (40.5)	15 097 (41.4)	0.66^h^
No	22 538 (58.6)	7011 (64.6)	15 527 (56.3)		22 538 (58.6)	1202 (59.5)	21 336 (58.6)	

Abbreviation: NA, not applicable.

^a^ One veteran was missing data for access to firearms.

^b^ *P* < .001.

^c^ The other race category includes American Indian/Pacific Islander, Asian, and unknown. The unknown race and ethnicity categories include veterans for whom this information is not disclosed or available.

^d^ Rural category includes both rural and highly rural Rural-Urban Commuting Area codes.

^e^ Acute Care group indicates veterans identified as intermediate or high acute risk of suicide in emergency department or urgent care settings and then discharged to go home.

^f^ Flagged group indicates veterans flagged by clinicians as being at particularly high risk for suicide.

^g^ *P* < .05.

^h^ *P* < .01.

### Reported Access to Firearms, Firearm Storage, and Related Interventions

Approximately one-third of included veterans (10 855 [28.2%]) reported access to firearms (Table 2). Among those reporting firearm access, 4406 (40.6%) stored at least 1 firearm locked and loaded, 2989 (27.5%) stored firearms unlocked and loaded, 1866 (17.2%) stored firearms locked and unloaded, 1185 (10.9%) stored firearms outside of the home, and 219 (2.0%) did not fall into any of these categories. Clinicians documented discussing firearm storage with 10 655 veterans (98.2%) with firearm access. Of those with firearm access, most veterans (8239 [75.9%]) declined a firearm lock or noted that they had another safe storage option already in place. Others accepted the firearm lock (1837 veterans [16.9%]), were not offered one (382 veterans [3.5%]), or requested one but none were available (241 veterans [2.2%]).

### Reported Access to Opioids and Related Interventions

Only 2021 veterans (5.3%) reported access to opioids (Table 2). Among veterans reporting access to opioids, clinicians documented discussing overdose risk with 1589 (78.6%) and requesting naloxone for 536 (26.5%). Other veterans declined naloxone (542 [26.8%]), noted that they already had it (370 [18.3%]), or responded in another way (550 [27.2%]).

### Sociodemographic Characteristics Associated With Access to Firearms and Opioids, Firearm Storage Practices, and Distribution of Firearm Cable Locks and Naloxone

Firearm access was more prevalent among veterans who were younger (age 18-39 years: OR, 1.22; 95% CI, 1.16-1.29; *P* < .001) and middle aged (age 40-54 years: OR, 1.22; 95% CI, 1.16-1.29; *P* < .001) vs older (age ≥55 years) (Table 3; eTable 1 in Supplement 1). Firearm access was also more prevalent among White veterans than Black veterans (OR, 1.41; 95% CI, 1.33-1.49; *P* < .001), veterans who were not Hispanic/Latine than Hispanic/Latine veterans (OR, 1.19; 95% CI, 1.09-1.29; *P* < .001), male veterans than female veterans (OR, 1.28; 95% CI, 1.20-1.36; *P* < .001), and those living in rural vs urban areas (OR, 1.75; 95% CI, 1.67-1.84; *P* < .001). Fewer veterans in the acute care (OR, 0.62; 95% CI, 0.57-0.68; *P* < .001), flagged (OR, 0.75; 95% CI, 0.71-0.79; *P* < .001), or both groups (OR, 0.71; 95% CI, 0.67-0.74; *P* < .001) reported access to firearms compared with veterans not in these groups. By contrast, younger (OR, 0.39; 95% CI, 0.34-0.43; *P* < .001) and middle-aged (OR, 0.62; 95% CI, 0.56-0.69; *P* < .001) veterans were less likely to have access to opioids than older veterans; the same was true for younger veterans compared with middle-aged veterans (OR, 0.62; 95% CI, 0.54-0.71; *P* < .001). White veterans, those who were not Hispanic/Latine, and rural veterans were more likely to have access to opioids than Black veterans (OR, 1.35; 95% CI, 1.20-1.50; *P* < .001), Hispanic/Latine veterans (OR, 1.56; 95% CI, 1.30-1.87; *P* < .001), and urban veterans (OR, 1.31; 95% CI, 1.19-1.45; *P* < .001), respectively. All results remained significant when controlling for other sociodemographic factors and veterans’ inclusion in the acute care or flagged groups.

We examined sociodemographic differences across veterans who reported storing their firearms outside of the home, locked in the home (including both loaded and unloaded), or unlocked in the home (Table 4; eTable 2 in Supplement 1). Younger and middle-aged veterans were less likely to report storing their firearms unlocked vs outside of the home than older veterans (younger: OR, 0.57; 95% CI, 0.48-0.67; *P* < .001; middle-aged: OR, 0.69; 95% CI, 0.58-0.82; *P* < .001); the same was true for younger vs middle-aged veterans (OR, 0.82; 95% CI, 0.69-0.98; *P* < .05). Older veterans were also less likely to report storing their firearms locked than unlocked compared with younger (OR, 0.58; 95% CI, 0.53,0.65; *P* < .001) and middle-aged (OR, 0.58; 95% CI, 0.52,0.65; *P* < .001) veterans. Additionally, male veterans were less likely to report storing their firearms unlocked or locked vs outside of the home than female veterans (unlocked: OR, 0.65; 95% CI, 0.52-0.81; *P* < .001; locked: OR, 0.66; 95% CI, 0.54-0.81; *P* < .001). Most differences remained significant when accounting for other sociodemographic variables and veterans’ inclusion in the acute care or flagged groups. We found no significant differences across race, ethnicity, or rurality, with the exception that veterans who were not Hispanic/Latine were more likely than Hispanic/Latine veterans to store their firearms unlocked vs locked (OR, 1.21; 95% CI, 1.03-1.44; *P* = .02).

**Table 4.** Demographic Differences Among Veterans at Elevated Risk for Suicide Based on Firearm Storage Patterns

Demographic group	Veterans by firearm storage, No. (%)					χ^2^
	Total (N = 10 446)	Outside of home (%) (n = 1185 [11.3%])	Locked and unloaded (n = 1866 [17.9%])	Locked and loaded (n = 4406 [42.2%])	Unlocked and loaded (n = 2989 [28.6])	
Age, y						
18-39	3678 (35.2)	467 (39.4)	707 (37.9)	1607 (36.5)	897 (30.0)	155.41^a^
40-54	3009 (28.8)	321 (27.1)	574 (30.8)	1366 (31.0)	748 (25.0)	
≥55	3759 (36.0)	397 (33.5)	585 (31.4)	1433 (32.5)	1344 (45.0)	
Sex						
Female	1435 (13.7)	116 (9.8)	230 (12.3)	660 (15.0)	429 (14.4)	25.42^b^
Male	9011 (86.3)	1069 (90.2)	1636 (87.7)	3746 (85.0)	2560 (85.7)	
Race						
Available data, No.	9576	1092	1696	4039	2749	
Black	2235 (23.3)	243 (22.3)	351 (20.7)	967 (23.9)	674 (24.5)	13.28^a^
White	6884 (71.9)	795 (72.8)	1253 (73.9)	2875 (71.2)	1961 (71.3)	
Other^c^	457 (4.8)	54 (5.0)	92 (5.4)	197 (4.9)	114 (4.2)	
Ethnicity						
Hispanic/Latine	810 (7.8)	90 (7.6)	167 (9.0)	339 (7.7)	214 (7.2)	39.18^b^
Not Hispanic/Latine	7773 (74.4)	884 (74.6)	1332 (71.4)	3220 (73.1)	2337 (78.2)	
Unknown^c^	1863 (17.8)	211 (17.8)	367 (19.7)	847 (19.2)	438 (14.7)	
Location						
Available data, No.	10 138	1137	1810	4280	2911	
Rural^d^	3484 (34.4)	376 (33.1)	642 (35.5)	1409 (32.9)	1057 (36.3)	10.67^a^
Urban	6654 (65.6)	761 (66.9)	1168 (64.5)	2871 (67.1)	1854 (63.7)	
Acute care group^e^						
Yes	605 (5.8)	86 (7.3)	89 (4.8)	256 (5.8)	174 (5.8)	8.25^a^
No	9841 (94.2)	1099 (92.7)	1777 (95.2)	4150 (94.2)	2815 (94.2)	
Flagged group^f^						
Yes	3311 (31.7)	525 (44.3)	455 (24.4)	1338 (30.4)	993 (33.2)	139.90^b^
No	7135 (68.3)	660 (55.7)	1411 (75.6)	3068 (69.6)	1996 (66.8)	
Acute care or flagged group^e^^,^^f^						
Yes	3705 (35.5)	575 (48.5)	514 (27.6)	1513 (34.3)	1103 (36.9)	144.55^b^
No	6741 (64.5)	610 (51.5)	1352 (72.5)	2893 (65.7)	1886 (63.1)	

^a^ *P* < .05.

^b^ *P* < .001.

^c^ The other race category includes American Indian/Pacific Islander, Asian, and unknown. The unknown race and ethnicity categories include veterans for whom this information is not disclosed or available.

^d^ Rural category includes both rural and highly rural Rural-Urban Commuting Area codes.

^e^ Acute Care group indicates veterans identified as intermediate or high acute risk of suicide in emergency department or urgent care settings and then discharged to go home.

^f^ Flagged group indicates veterans flagged by clinicians as being at particularly high risk for suicide.

Lastly, we examined sociodemographic differences across veterans depending on documentation of firearm lock and naloxone distribution (Table 5; eTable 3 in Supplement 1). Middle-aged veterans were less likely to accept firearm locks than older veterans (OR, 0.84; 95% CI, 0.74-0.96; *P* = .008). Black veterans, female veterans, and veterans who were not Hispanic/Latine were less likely to accept gunlocks than White (OR, 0.57; 95% CI, 0.50-0.64; *P* < .001), male (OR, 0.84; 95% CI, 0.73-0.97; *P* = .02), and Hispanic/Latine (OR, 0.83; 95% CI, 0.69,0.99; *P* = .047) veterans. More veterans in the flagged group (OR, 1.27; 95% CI, 1.14-1.41; *P* < .001) or both the flagged and acute care groups (OR, 1.25; 95% CI, 1.13-1.39; *P* < .001) accepted firearm locks compared with veterans not in these groups. Most differences remained significant when controlling for other sociodemographic factors and veterans’ inclusion in the acute care or flagged groups, except that differences between female and male veterans lost significance. We found no sociodemographic differences for naloxone distribution. More veterans in the acute care (OR, 2.05; 95% CI, 1.25-3.34; *P* = .004), flagged (OR, 1.29; 95% CI, 1.00-1.65; *P* = .046), or both (OR, 1.44; 95% CI, 1.13-1.84; *P* = .003) groups were documented as accepting naloxone compared with veterans not in these groups.

**Table 5.** Demographic Differences Among Veterans Who Did and Did Not Receive Firearm Locks and Naloxone

Demographic group	Offered gunlock				Offered naloxone			
	Veterans, No. (%)			χ^2^	Veterans, No. (%)			χ^2^
	Total (n = 10 074)^a^	Provided (n = 1837 [18.2%])	Declined (n = 8237 [81.7%])		Total (n = 1079)	Provided (n = 536 [49.7%])	Declined (n = 543 [50.3%])	
Age, y								
18-39	3545 (35.2)	642 (35.0)	2903 (35.2)	7.2^b^	218 (20.2)	107 (20.0)	111 (20.4)	0.6
40-54	2892 (28.7)	488 (26.6)	2404 (29.2)		260 (24.1)	136 (25.4)	124 (22.8)	
≥55	3637 (36.1)	707 (38.5)	2930 (35.6)		601 (55.7)	293 (54.7)	308 (56.7)	
Sex								
Female	1360 (13.5)	280 (15.2)	1080 (13.1)	5.8^b^	179 (16.6)	92 (17.2)	87 (16.0)	0.3
Male	8714 (86.5)	1557 (84.8)	7157 (86.9)		900 (83.4)	444 (82.8)	456 (84.0)	
Race								
Available data, No.	9235	1679	7556		1002	494	508	
Black	2140 (23.2)	536 (31.9)	1604 (21.2)	91.8^d^	236 (23.6)	116 (23.5)	120 (23.6)	0.4
White	6658 (72.1)	1058 (63.0)	5600 (74.1)		718 (71.7)	355 (71.9)	363 (71.5)	
Other^c^	437 (4.7)	85 (5.1)	352 (4.7)		48 (4.8)	23 (4.7)	25 (4.92)	
Ethnicity								
Hispanic/Latine	785 (7.8)	162 (8.8)	623 (7.6)	5.1	68 (6.3)	40 (7.5)	28 (5.2)	6.5^b^
Not Hispanic/Latine	7491 (74.4)	1331 (72.5)	6160 (74.8)		890 (82.5)	447 (83.4)	443 (81.6)	
Unknown^c^	1798 (18.7)	344 (18.7)	1454 (17.7)		121 (11.2)	49 (9.1)	72 (13.3)	
Location								
Available data, No.	9776	1788	7988		1034	512	522	
Rural^e^	3377 (34.5)	592 (33.1)	2785 (34.9)	2.0	331 (32.0)	157 (30.7)	174 (33.3)	0.8
Urban	6399 (65.5)	1196 (66.9)	5203 (65.1)		703 (68.0)	355 (69.3)	348 (66.7)	
Acute care group^f^								
Yes	578 (5.7)	94 (5.1)	484 (5.9)	1.6	76 (7.0)	50 (9.3)	26 (4.8)	8.5^g^
No	9496 (94.3)	1743 (94.9)	7753 (94.1)		1003 (93.0)	486 (90.7)	517 (95.2)	
Flagged group^h^								
Yes	3194 (31.7)	661 (36.0)	2533 (30.8)	19.0^d^	395 (36.6)	212 (39.6)	183 (33.7)	4.0^b^
No	6880 (68.3)	1176 (64.0)	5704 (69.3)		684 (63.4)	324 (60.5)	360 (66.3)	
Acute care or flagged group^f^^,^^h^								
Yes	3571 (35.5)	729 (40.0)	2842 (34.5)	17.6^g^	443 (41.1)	244 (45.5)	199 (36.7)	8.8^d^
No	6503 (64.6)	1108 (60.0)	5395 (65.5)		636 (58.9)	292 (54.5)	344 (63.4)	

^a^ One veteran was missing data for access to firearms.

^b^ *P* < .05.

^c^ The other race category includes American Indian/Pacific Islander, Asian, and unknown. The unknown race and ethnicity categories include veterans for whom this information is not disclosed or available.

^d^ *P* < .01.

^e^ Rural category includes both rural and highly rural Rural-Urban Commuting Area codes.

^f^ Acute Care group indicates veterans identified as intermediate or high acute risk of suicide in emergency department or urgent care settings and then discharged to go home.

^g^ *P* < .001.

^h^ Flagged group indicates veterans flagged by clinicians as being at particularly high risk for suicide.

## Discussion

This cross-sectional study is the first to describe access to lethal means and related interventions among a national sample of veterans at elevated risk for suicide, providing information critical to improving lethal means safety efforts in the VHA and other large health systems. Encouraging individuals at risk for suicide to limit their access to lethal means, especially firearms and substances that can be used for overdose, is a key component of evidence-based suicide prevention programs. The VHA has implemented a nationwide program to deliver LMC to veterans with elevated suicide risk through suicide safety planning and other interventions.

We found that among veterans at elevated risk for suicide who completed suicide safety plans, 28% reported access to firearms. Approximately one-third of these veterans reported storing at least 1 firearm in the following ways: unlocked and loaded (insecure), outside of the home or locked and unloaded (secure) or locked and loaded. A small proportion (17%) of veterans with access to firearms were documented as accepting a gunlock, with most declining one or noting that they already stored their firearms securely. Younger and middle-aged veterans and White veterans, those who were not Hispanic/Latine, male veterans, and rural veterans reported more access to firearms while veterans in the acute care or flagged groups reported less access to firearms. Older veterans were more likely to report storing their firearms unlocked than locked or outside of the home. Female veterans were less likely to report storing their firearms outside of the home than locked or unlocked. Black veterans, female veterans, and veterans who were not Hispanic/Latine were less likely to accept firearm cable locks, while veterans in the flagged group were more likely to accept them. Only 5% of veterans who completed safety plans reported access to opioids, with approximately one-third documented as accepting naloxone. Older veterans, White veterans, veterans who were not Hispanic/Latine, and rural veterans were more likely to report having access to opioids. Veterans in the acute care or flagged groups were more likely to accept naloxone.

While some of these data are consistent with our hypotheses and patterns found in previous research,^9,10^ others appear to be unique to veterans with elevated suicide risk receiving LMC from their VHA clinicians. For example, previous studies have found that approximately one-half of veterans in the general population report access to firearms,^6,7,8^ while only 28% of veterans in our sample reported firearm access. Lower levels of firearm access in our sample suggest underreporting among veterans, underdocumentation among clinicians during clinical encounters, or a true lower prevalence among this higher-risk population.

Potential underreporting of firearm access is supported by previous research showing that veterans and nonveterans may be reluctant to disclose firearm ownership when discussing suicide risk because of fears that their firearms may be confiscated or that disclosing firearm access might impact their care.^25,26,27^ As safety planning can be completed with clinicians with whom veterans do not have an established relationship, it is possible that some veterans may not have disclosed firearm access due to discomfort.^27,28,29^ Interestingly, when surveyed directly, many veterans support clinicians asking about firearm access in the context of elevated suicide risk.^30,31^ Furthermore, training clinicians to use strategies that increase the acceptability of LMC (eg, contextualizing LMC and providing a rationale) may help them earn patients’ trust.^32,33,34^ It is also possible that clinicians underdocumented firearm access, perhaps due to veterans’ concerns about documentation of firearm ownership in their clinical records.^31^ In some cases, veterans with identified suicide risk receiving mental health care may have already made efforts to limit their firearms access. This is especially the case for veterans flagged as being at particularly high risk for suicide, who reported lower levels of firearm access than the rest of the sample. Overall, this research highlights the importance of disseminating accurate information on the rationale for clinicians discussing and documenting firearm access and storage with patients to help prevent suicide, and the rare potential consequences of doing so (eg, the Department of Veterans Affairs (VA) “Correcting Mistaken Beliefs about the VA Confiscating Veterans’ Guns” handout^35^).

Consistent with previous research, we found that one-third of veterans stored at least 1 firearm insecurely.^8,9,10^ We also found that only a small proportion of veterans (17%) accepted no-cost firearm cable locks, perhaps in part due to firearm owners’ preferences for higher-quality and more expensive devices, like lockboxes.^36,37^ These findings provide support for new efforts within the VHA to make no-cost firearm lockboxes available to veterans at elevated risk for suicide.

Additionally, we found that 5% of included veterans reported access to opioids, similar to estimates in 2020 that 7% of veterans with VHA pharmacy data were prescribed opioids (down from 17% in 2012).^13^ Importantly, information in the safety plan does not distinguish between access to prescribed and nonprescribed opioids (eg, fentanyl, nonprescribed pills). Furthermore, veterans may have underreported access to opioids when discussing elevated suicide risk due to concerns that their access to prescribed opioids would be limited.^11^ While we also found that only approximately one-third of veterans with access to opioids agreed to accept naloxone, 18% indicated that they already had it, perhaps in part due to the VHA’s efforts to distribute naloxone to all patients with opioid access and parallel efforts in community and non-VHA settings.^11,13^

We also found that discussion of firearm storage and overdose risk was provided to most veterans with access to these means. These numbers suggest that prompting clinicians via a standardized note template to complete LMC may have resulted in more clinicians having these discussions. However, research is needed to examine the quality of template-prompted LMC. Research is also needed to find strategies to encourage veterans to accept and use firearm locks and naloxone.

Consistent with previous work, we found that male and rural veterans were more likely to report access to firearms^6,7,9,38^ and that White veterans, those who were not Hispanic/Latine, and rural veterans were more likely to report access to opioids.^16,17^ These and other demographic differences observed suggest that clinicians should consider patients’ sociodemographic backgrounds when conducting LMC.^28,39^

### Limitations

This study should be considered alongside its limitations. Most importantly, our data reflect self-reported information as recorded by clinicians during safety planning, the validity of which has not been determined. Furthermore, EHR data may contain errors, especially as clinicians were required to respond to each prompt in the safety plan to complete the note template. In particular, firearm storage options were assessed by having clinicians check all storage options applicable to each veteran, which resulted in some inconsistent responding and required that we hierarchically categorize the data by prioritizing the most secure storage option. Furthermore, given our interest in veterans identified as being at elevated suicide risk on a standardized suicide risk assessment, we did not include information from safety plans completed in other contexts and among other patient populations that may have yielded different results. Because information about access to firearms and opioids is not available for all veterans at elevated risk for suicide, we focused on veterans who completed safety planning. However, these veterans may differ from other veterans who were not offered safety plans or refused to complete one. Similarly, veterans at elevated risk for suicide seeking care in the VHA likely differ from veterans who do not receive VHA health care. Furthermore, while our large sample size was a strength, we were also overpowered to detect small differences in sociodemographic characteristics.

## Conclusions

In this cross-sectional study of VHA EHR data, we found lower than expected prevalence of reported access to firearms among veterans with elevated suicide risk receiving safety planning, with only one-third of these veterans reporting at least 1 securely stored firearm. A small proportion of veterans reported access to opioids, and only one-third of these veterans accepted naloxone. Completion of a note template may have encouraged routine discussion of firearm storage and overdose risk, but further work is needed to understand the validity and quality of these interactions. Our findings suggest that large health care systems can systematically identify patients with risk factors for suicide to facilitate implementation of targeted interventions. Variations across patient groups indicates the need to assess for, understand, and address disparities in care. Importantly, there may be a need to educate clinicians on strategies to increase the acceptability of LMC and disseminate accurate information to patients on the rationale and consequences for discussing firearm access as a suicide prevention strategy.

## Notes

Supplement 1. **eAppendix.** C-SSRS Screen **eFigure.** Patient Recruitment Flowchart **eTable 1.** Demographic Differences Among Veterans With and Without Access to Firearms and Opioids **eTable 2.** Demographic Differences Among Veterans at Elevated Risk for Suicide Depending on Firearm Storage Patterns **eTable 3.** Demographic Differences Among Veterans Who Did and Did Not Accept Firearm Locks and Naloxone

Supplement 2.
Data Sharing Statement
