# Supplementary material for: Access to Firearms and Opioids Among Veterans at Risk for Suicide
Source: JAMA Netw Open. 2025 Jan 28;8(1):e2456906. doi: 10.1001/jamanetworkopen.2024.56906 (PMC11775732; doi:10.1001/jamanetworkopen.2024.56906)
Supplement: Supplement 1. — eAppendix. C-SSRS Screen eFigure. Patient Recruitment Flowchart eTable 1. Demographic Differences Among Veterans With and Without Access to Firearms and Opioids eTable 2. Demographic Differences Among Veterans at Elevated Risk for Suicide Depending on Firearm Storage Patterns eTable 3. Demographic Differences Among Veterans Who Did and Did Not Accept Firearm Locks and Naloxone [file jamanetwopen-e2456906-s001.pdf]

Supplemental Online Content

Khazanov GK, Wilson M, Cidav T, et al. Access to firearms and opioids among veterans at risk for suicide. *JAMA Netw Open*. 2025;8(1):e2456906. doi:10.1001/jamanetworkopen.2024.56906

eAppendix. C-SSRS Screen

eFigure. Patient Recruitment Flowchart

eTable 1. Demographic Differences Among Veterans With and Without Access to Firearms and Opioids

eTable 2. Demographic Differences Among Veterans at Elevated Risk for Suicide Depending on Firearm Storage Patterns

eTable 3. Demographic Differences Among Veterans Who Did and Did Not Accept Firearm Locks and Naloxone

This supplemental material has been provided by the authors to give readers additional information about their work.

© 2025 Khazanov GK et al. *JAMA Network Open*.

eAppendix 1

C-SSRS Screen

1. Over the past month, have you wished you were dead or wished you could go to sleep and not wake up?
   - ☐ Yes
   - ☐ No

   Proceed to question #2 regardless of response.

2. Over the past month, have you had any actual thoughts of killing yourself?
   - ☐ Yes
   - ☐ No

   If 'Yes', proceed to question #3

   If 'No', proceed to question #7

3. Over the past month, have you been thinking about how you might do this?
   - ☐ Yes
   - ☐ No

   Proceed to question #4 regardless of response.

4. Over the past month, have you had these thoughts and had some intention of acting on them?
   - ☐ Yes
   - ☐ No

   Proceed to question #5 regardless of response.

5. Over the past month, have you started to work out or worked out the details of how to kill yourself?
   - ☐ Yes
   - ☐ No

   If 'Yes', proceed to question #6

   If 'No', proceed to question #7

6. If yes to Q5, at any time in the past month did you intend to carry out this plan?
   - ☐ Yes
   - ☐ No

   Proceed to question #7 regardless of response.

7. In your lifetime, have you ever done anything, started to do anything, or prepared to do anything to end your life (for example, collected pills, obtained a gun, gave away valuables, went to the roof but didn't jump)?
   - ☐ Yes
   - ☐ No

   If 'Yes', proceed to question #8

   If 'No', proceed to scoring

8. If yes to Q7, was this within the past 3 months?
   - ☐ Yes
   - ☐ No

   Proceed to scoring

Scoring: A positive C-SSRS (Columbia) score is a 'Yes' response to items 3, 4, 5, or 8.

© 2025 Khazanov GK et al. *JAMA Network Open*.

eFigure 1.

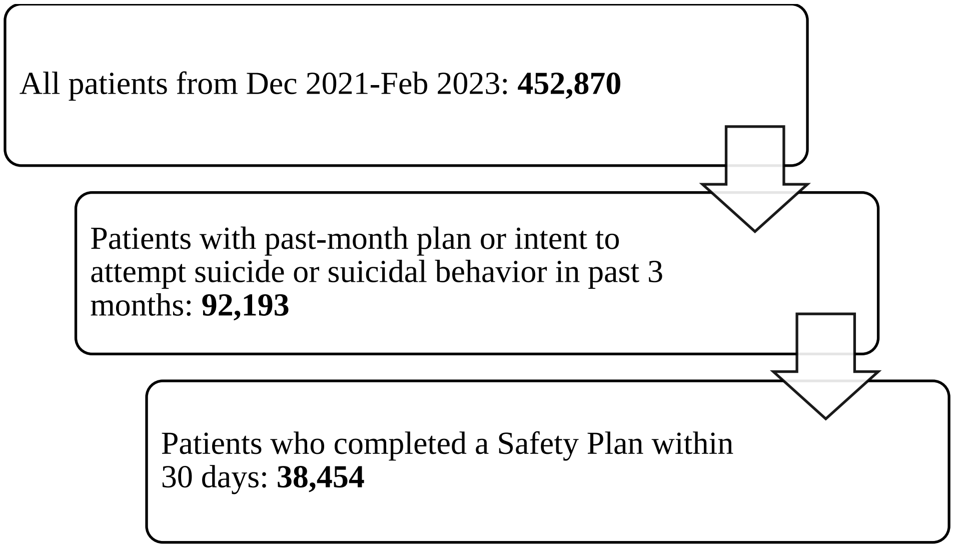

© 2025 Khazanov GK et al. *JAMA Network Open*.

eTable 1. Demographic differences among Veterans with and without access to firearms and opioids – odds ratios

	Access to Firearms			Access to Opioids		
	No controls	Model 1	Model 2	No controls	Model 1	Model 2
Group	Odds ratio (95% CI)	Odds ratio (95% CI)	Odds ratio (95% CI)	Odds ratio (95% CI)	Odds ratio (95% CI)	Odds ratio (95% CI)
**Age** (reference category=55+)						
18-39 vs 55+	1.22(1.16,1.29) ***	1.26 (1.19,1.33) ***	1.29 (1.21,1.36) ***	0.39 (0.34,0.43) ***	0.38 (0.34,0.43) ***	0.38 (0.33,0.43) ***
40-54 vs 55+	1.22 (1.16,1.29) ***	1.23 (1.16,1.31) ***	1.25 (1.17,1.32) ***	0.62 (0.56,0.69) ***	0.58 (0.52,0.65) ***	0.58 (0.52,0.65) ***
**Age** (reference category=18-39)						
40-54 vs 18-39	1.00 (0.95,1.06)	0.98 (0.92,1.04)	0.97 (0.91,1.03)	[a]1.61 (1.41,1.85) ***	1.53 (1.32,1.77) ***	1.53 (1.33,1.77) ***
55+ vs 18-39	0.82 (0.78,0.86) ***	0.80 (0.75,0.84) ***	0.78 (0.74,0.82) ***	2.60 (2.31,2.92) ***	2.63 (2.32,2.99) ***	2.64 (2.32,2.99) ***
**Race**						
Black vs. White	[b]0.71 (0.67,0.75) ***	0.80 (0.76,0.85) ***	0.79 (0.75,0.84) ***	[c]0.74 (0.67,0.83) ***	0.73 (0.65,0.82) ***	0.73 (0.65,0.82) ***
Other vs. White	0.83 (0.74,0.92) ***	0.86 (0.77,0.96) **	0.84 (0.75,0.94) **	0.70 (0.55,0.88) **	0.76 (0.59,0.98) *	0.77 (0.59,0.98) *
**Gender**						
Male vs Female	1.28 (1.20,1.36) ***	1.27 (1.78,1.36) ***	1.28 (1.19,1.37) ***	0.94 (0.83,1.06)	0.77 (0.67,0.87) ***	0.77 (0.67,0.87) ***
**Ethnicity** (reference category = Hispanic/Latine)						
Not Hispanic/Latine	1.19 (1.09,1.29) ***			1.56 (1.30,1.87) ***		
Unknown	1.42 (1.29,1.56) ***			0.85 (0.68,1.06)		
**Location**						
Rural vs Urban	1.75 (1.67,1.84) ***	1.68 (1.60,1.77) ***	1.68 (1.60,1.77) ***	1.31 (1.19,1.45) ***	1.23 (1.10,1.36) ***	1.23 (1.10,1.36) ***
**Acute Care Group**						
Yes vs. No	0.62 (0.57,0.68) ***			1.01 (0.86,1.19)		
**Flagged Group**						
Yes, vs No	0.75 (0.71,0.79)			0.693		

© 2025 Khazanov GK et al. *JAMA Network Open*.

	***					
**Acute Care or Flagged Group**						
Yes vs. No	0.71 (0.67,0.74) ***		0.69 (0.66,0.72) ***	0.96 (0.88,1.06)		1.01 (0.91,1.11)

Note. Numbers represent demographic differences among veterans who received Safety Plans within 30 days of being identified as at-risk for suicide depending on their access to firearms and opioids. CI = Confidence Interval. Model 1 = Controlling for sociodemographics (age, race, gender, location); Model 2 = Controlling for sociodemographics and inclusion in the Acute Care or Flagged Groups. CI = Confidence Interval. The “other” race category includes Asian, American Indian/Pacific Islander, and Unknown. The “unknown” race and ethnicity categories include veterans for whom this information is not disclosed or available. Rural category includes both Rural and Highly Rural RUCA codes. Acute Care group = veterans identified as intermediate or high acute risk of suicide and then discharged to go home; Flagged group = veterans flagged by providers as being at particularly high risk for suicide. Rural category includes both Rural and Highly Rural RUCA codes.

[a] The number reported in the text uses middle-aged veterans as the reference category (18-39 vs. 40-45 = 0.62 (0.54, 0.71)***)

[b] The number reported in the text uses Black as the reference category (White vs. Black = 1.41 (1.33, 1.49)***)

[c] The number reported in the text uses Black as the reference category (White vs. Black = 1.35 (1.20, 1.50)***)

*p < .05, **p<.01, ***p<.001

© 2025 Khazanov GK et al. *JAMA Network Open*.

eTable 2. Demographic differences among Veterans at elevated risk for suicide depending on firearm storage patterns (odds ratios).

		No controls	Model 1	Model 2		No controls	Model 1	Model 2
Variable	Reference category = Outside of home	Odds ratio (95% CI)	Odds ratio (95% CI)	Odds ratio (95% CI)	Reference category = Locked	Odds ratio (95% CI)	Odds ratio (95% CI)	Odds ratio (95% CI)
**Age** (reference category=55+)								
18-39 vs 55+	Unlocked	0.57 (0.48,0.67) ***	0.55 (0.46,0.65) ***	0.57 (0.48,0.68) ***	Unlocked	[a]0.58 (0.53,0.65) ***	0.59 (0.53,0.66) ***	0.58 (0.52,0.65) ***
18-39 vs 55+	Locked	0.98 (0.84,1.13)	0.93 (0.79,1.09)	0.98 (0.84,1.15)	Outside of home	1.03 (0.89,1.19)	1.08 (0.92,1.27)	1.02 (0.87,1.20)
40-54 vs 55+	Unlocked	0.69 (0.58,0.82) ***	0.66 (0.55,0.79) ***	0.67 (0.56,0.81) ***	Unlocked	[b]0.58 (0.52,0.65) ***	0.57 (0.51,0.64) ***	0.57 (0.51,0.64) ***
40-54 vs 55+	Locked	1.19 (1.01,1.40) *	1.15 (0.97,1.36)	1.18 (1.00,1.33)	Outside of home	0.84 (0.72,0.99) *	0.87 (0.74,1.03)	0.85 (0.71,1.00)
**Age** (reference category=18-39)								
40-54 vs 18-39	Unlocked	[c]1.21 (1.02,1.44) *	1.20 (1.00,1.45)	1.18 (0.98,1.42)	Unlocked	1.00 (0.89,1.12)	0.97 (0.86,1.10)	0.98 (0.87,1.10)
40-54 vs 18-39	Locked	1.22 (1.05,1.42) *	1.24 (1.05,1.46) *	1.21 (1.02,1.43) *	Outside of home	0.82 (0.70,0.96) *	0.81 (0.69,0.95) *	0.83 (0.70,0.98) *
55+ vs 18-39	Unlocked	1.76 (1.51,2.07) ***	1.83 (1.54,2.17) ***	1.76 (1.48,2.09) ***	Unlocked	1.72 (1.55,1.91) ***	1.69 (1.51,1.89) ***	1.72 (1.54,1.93) ***
55+ vs 18-39	Locked	1.03 (0.89,1.19)	1.08 (0.92,1.27)	1.02 (0.87,1.20)	Outside of home	0.98 (0.84,1.13)	0.93 (0.79,1.09)	0.98 (0.84,1.15)
**Race**								
Black vs. White	Unlocked	1.12 (0.95,1.33)	1.10 (0.92,1.31)	1.11 (0.93,1.33)	Unlocked	1.08 (0.97,1.20)	1.06 (0.95,1.19)	1.06 (0.94,1.18)
Black vs. White	Locked	1.05 (0.89,1.22)	1.04 (0.88,1.22)	1.05 (0.89,1.24)	Outside of home	0.96 (0.82,1.12)	0.96 (0.82,1.14)	0.95 (0.81,1.12)
Other vs. White	Unlocked	0.86 (0.61,1.20)	0.97 (0.68,1.37)	0.96 (0.68,1.36)	Unlocked	0.83 (0.66,1.04)	0.92 (0.73,1.01)	0.93 (0.74,1.17)
Other vs. White	Locked	1.03 (0.76,1.39)	1.05 (0.77,1.43)	1.04 (0.76,1.42)	Outside of home	0.97 (0.72,1.31)	0.96 (0.70,1.31)	0.97 (0.70,1.32)
**Gender**								
Male vs. Female	Unlocked	0.65 (0.52,0.81) ***	0.62 (0.49,0.78) ***	0.62 (0.49,0.78) ***	Unlocked	0.99 (0.87,1.12)	0.88 (0.77,1.01)	0.88 (0.77,1.01)
Male vs. Female	Locked	0.66 (0.54,0.81) ***	0.70 (0.56,0.87) **	0.70 (0.56,0.87) **	Outside of home	1.52 (1.24,1.87) ***	1.43 (1.15,1.77) **	1.43 (1.15,1.78) **
**Ethnicity** (reference category = Hispanic/Latine)								

© 2025 Khazanov GK et al. *JAMA Network Open*.

Not Hispanic or Latino	Unlocked	1.11 (0.86,1.44)			Unlocked	1.21 (1.03,1.44) *		
Not Hispanic or Latino	Locked	0.92 (0.72,1.16)			Outside of home	1.09 (0.86,1.38)		
Unknown	Unlocked	0.87 (0.65,1.17)			Unlocked	0.85 (0.70,1.04)		
Unknown	Locked	1.02 (0.78,1.34)			Outside of home	0.98 (0.75,1.28)		
**Location**								
Rural vs. Urban	Unlocked	1.15 (1.00,1.33)	1.21 (1.03,1.41) *	1.20 (1.03.1.40) *	Unlocked	1.15 (1.00,1.33)	1.10 (1.00,1.22)	1.11 (1.00,1.22) *
Rural vs. Urban	Locked	1.03 (0.90,1.18)	1.09 (0.95,1.26)	1.09 (0.94,1.26)	Outside of home	1.03 (0.90,1.18)	0.91 (0.79,1.06)	0.92 (0.80,1.06)
**Acute Care Group**								
Yes vs. No	Unlocked	0.79 (0.60,1.03)			Unlocked	1.06 (0.88,1.28)		
Yes vs. No	Locked	0.74 (0.58,0.95) *			Outside of home	1.35 (1.05,1.72) *		
**Flagged Group**								
Yes vs. No	Unlocked	0.63 (0.55,0.72) ***			Unlocked	1.24 (1.13,1.37) ***		
Yes vs. No	Locked	0.50 (0.44,0.57) ***			Outside of home	1.99 (1.75,2.26) ***		
**Acute Care or Flagged Group**								***
Yes vs. No	Unlocked	0.62 (0.54,0.71) ***		0.64 (0.55,0.74) ***	Unlocked	1.23 (1.12,1.34) ***		1.24 (1.12,1.36) ***
Yes vs. No	Locked	0.51 (0.45,0.57) ***		0.52 (0.45,0.59) ***	Outside of home	1.97 (1.74,2.24) ***		1.94 (1.70,2.22) ***

Note. Numbers represent demographic differences among veterans who received Safety Plans within 30 days of being identified as at-risk for suicide depending on their storage of firearms. Locked category includes both loaded/locked and unloaded/locked. Numbers do not include the “other” firearm storage category. CI = Confidence Interval. Model 1 = Controlling for sociodemographics (age, race, gender, location); Model 2 = Controlling for sociodemographics and inclusion in the Acute Care or Flagged Groups. The “other” race category includes Asian, American Indian/Pacific Islander, and Unknown. The “unknown” race and ethnicity categories include veterans for whom this information is not disclosed or available. Rural category includes both Rural and Highly Rural RUCA codes. Acute Care group = veterans identified as intermediate or high acute risk of suicide and then discharged to go home; Flagged group = veterans flagged by providers as being at particularly high risk for suicide. Rural category includes both Rural and Highly Rural RUCA codes.

[a] The number reported in the text uses young veterans and unlocked as the reference categories; results are identical

© 2025 Khazanov GK et al. *JAMA Network Open*.

[b] The number reported in the text uses middle aged veterans and unlocked as the reference categories; results are identical
[c] The number reported in the text uses middle-aged veterans as the reference category (18-39 vs. 40-54 = 0.82 (0.69, 0.98)*)
*p < .05, **p<.01, ***p<.001

© 2025 Khazanov GK et al. *JAMA Network Open*.

eTable 3. Demographic differences among Veterans who did and did not accept firearm locks and naloxone – odds ratios

	Accepted Gunlock			Accepted Naloxone		
	No controls	Model 1	Model 2	No controls	Model 1	Model 2
Group	Odds ratio (95% CI)	Odds ratio (95% CI)	Odds ratio (95% CI)	Odds ratio (95% CI)	Odds ratio (95% CI)	Odds ratio (95% CI)
**Age** (reference category=55+)						
18-39 vs 55+	0.92(0.81,1.03)	0.95 (0.84,1.08)	0.93 (0.82,1.06)	1.01 (0.74,1.38)	1.09 (0.78,1.52)	1.07 (0.76,1.49)
40-54 vs 55+	0.84 (0.74,0.96) **	0.84 (0.74,0.97) *	0.84 (0.73,0.96) **	1.15 (0.86,1.54)	1.15 (0.84,1.58)	1.15 (0.84,1.57)
**Age** (reference category=18-39)						
40-54 vs 18-39	0.92 (0.81,1.05)	0.89 (0.77,1.02)	0.89 (0.78,1.03)	1.14 (0.79,1.63)	1.06 (0.72,1.56)	1.07 (0.73,1.58)
55+ vs 18-39	1.09 (0.97,1.23)	1.05 (0.93,1.20)	1.07 (0.94,1.22)	0.99 (0.72,1.35)	0.92 (0.66,1.28)	0.94 (0.67,1.31)
**Race**						
Black vs. White	[a]1.77 (1.57,1.99) ***	1.77 (1.57,2.01) ***	1.77 (1.56,2.00) ***	0.99 (0.74,1.33)	0.96 (0.70,1.30)	0.97 (0.71,1.32)
Other vs. White	1.28 (1.00,1.63)	1.26 (0.97,1.62)	1.26 (0.98,1.63)	0.94 (0.52,1.69)	0.84 (0.45,1.58)	0.83 (0.44,1.55)
**Gender**						
Male vs Female	0.84 (0.73,0.97) *	0.92 (0.79,1.08)	0.92 (0.79,1.08)	0.92 (0.67,1.27)	0.86 (0.61,1.22)	0.88 (0.62,1.24)
**Ethnicity** (reference category = Hispanic/Latine)						
Not Hispanic/Latine	0.83 (0.69,0.99) *			0.71 (0.43,1.17)		
Unknown	0.91 (0.74,1.12)			0.48 (0.26,0.87) *		
**Location**						
Rural vs Urban	0.93 (0.83,1.03)	1.01 (0.90,1.14)	1.02 (0.90,1.14)	0.89 (0.68,1.15)	0.90 (0.68,1.19)	0.89 (0.67,1.17)
**Acute Care Group**						
Yes vs. No	0.86 (0.69,1.08)			2.05 (1.25,3.34) **		
**Flagged Group**						
Yes, vs No	1.27 (1.14,1.41) ***			1.29 (1.00,1.65) *		

© 2025 Khazanov GK et al. *JAMA Network Open*.

**Acute Care or Flagged Group**						
Yes vs. No	1.25 (1.13,1.39) ***		1.25 (1.12,1.40) ***	1.44 (1.13,1.84) **		1.47 (1.14,1.91) **

Note. Numbers represent demographic differences among veterans who received Safety Plans within 30 days of being identified as at-risk for suicide depending on their acceptance of gunlocks and naloxone. For gunlocks, we only included veterans who were provided gunlocks (n = 1,837) versus those who declined them (n = 8,239); we did not include veterans who were not offered gunlocks (n = 382), requested gunlocks but none were available (n = 241), or had missing data. For naloxone, we only included veterans for whom providers ordered naloxone (n = 536) versus those who declined naloxone (n = 543); we did not include veterans who already had naloxone (n = 370) or had other/missing data (n = 573). CI = Confidence Interval. Model 1 = Controlling for sociodemographics (age, race, gender, location); Model 2 = Controlling for sociodemographics and inclusion in the Acute Care or Flagged Groups. The “other” race category includes Asian, American Indian/Pacific Islander, and Unknown. The “unknown” race and ethnicity categories include veterans for whom this information is not disclosed or available. Rural category includes both Rural and Highly Rural RUCA codes. Acute Care group = veterans identified as intermediate or high acute risk of suicide and then discharged to go home; Flagged group = veterans flagged by providers as being at particularly high risk for suicide.

[a] The number reported in the text uses Black as the reference category (White vs. Black = 0.57 (0.50, 0.64)***)

*p < .05, **p<.01, ***p<.001

© 2025 Khazanov GK et al. *JAMA Network Open*.
