# Supplementary material for: Access to Firearms and Opioids Among Veterans at Risk for Suicide
Source: JAMA Netw Open. 2025 Jan 28;8(1):e2456906. doi: 10.1001/jamanetworkopen.2024.56906 (PMC11775732; doi:10.1001/jamanetworkopen.2024.56906)
Supplement: Supplement 2. — Data Sharing Statement [file jamanetwopen-e2456906-s002.pdf]

Data Sharing Statement

Khazanov. Access to Firearms and Opioids Among Veterans at Risk for Suicide. *JAMA Netw Open*. Published January 28, 2025. doi:10.1001/jamanetworkopen.2024.56906

Data
Data available: No

Additional Information
Explanation for why data not available: We do not have permission to share confidential health records.
